# LRRC3B and its promoter hypomethylation status predicts response to anti-PD-1 based immunotherapy

**DOI:** 10.3389/fimmu.2023.959868

**Published:** 2023-01-24

**Authors:** Linfeng Luo, Sha Fu, Wei Du, Li-na He, Xuanye Zhang, Yixing Wang, Yixin Zhou, Shaodong Hong

**Affiliations:** ^1^ State Key Laboratory of Oncology in South China, Sun Yat-sen University Cancer Center, Guangzhou, China; ^2^ Collaborative Innovation Center for Cancer Medicine, Sun Yat-sen University Cancer Center, Guangzhou, China; ^3^ Department of Medical Oncology, Sun Yat-sen University Cancer Center, Guangzhou, China; ^4^ Department of Cellular & Molecular Diagnostics Center, Sun Yat-Sen Memorial Hospital, Sun Yat-Sen University, Guangzhou, China; ^5^ Guangdong Provincial Key Laboratory of Malignant Tumor Epigenetics and Gene Regulation of Sun Yat-Sen University, Guangzhou, China; ^6^ Department of VIP Region, Sun Yat-sen University Cancer Center, Guangzhou, China

**Keywords:** pan-cancer, tumor immune microenvironment, immunotherapy, leucine rich repeat containing 3B, DNA methylation

## Abstract

**Background:**

The leucine rich repeat containing 3B (LRRC3B) gene is a tumor suppressor gene involved in the anti-tumor immune microenvironment. Expression of LRRC3B and DNA methylation at the LRRC3B promoter region may serve as a useful marker to predict response to anti-PD-1 therapy. However, no studies have yet systematically explored the protective role of LRRC3B methylation in tumor progression and immunity.

**Methods:**

Expression of LRRC3B of 33 cancer types in The Cancer Genome Atlas (TCGA) was downloaded from UCSC Xena (http://xena.ucsc.edu/). And, we evaluated the differential expression of LRRC3B according to tumor stage, overall survival, and characteristics of the tumor microenvironment. The immunotherapeutic cohorts included IMvigor21, GSE119144, and GSE72308 which were obtained from the Gene Expression Omnibus database. We conducted pearson correlation analysis of LRRC3B and tumor microenvironment (TME) in pan-cancer. Also, six immune cell types (B cells, CD8+ T cells, CD4+ T cells, macrophages, neutrophils, and dendritic cells) and tumor purity were analyzed using the Tumor IMmune Estimation Resource (TIMER1.0) (Tumor IMmune Estimation Resource (TIMER2.0). And, a “silencing score” model base on LRRC3B promoter methylation to predict overall survival (OS) by multivariate Cox regression analysis was constructed. Finally, the model was applied to predict anti-PD-1 therapy in non-small cell lung cancer (NSCLC) and breast cancer (BRCA).

**Results:**

LRRC3B expression associated with less tumor invasion, less severe tumor stage, and decreased metastasis. The inactivation of LRRC3B promoted the enrichment of immuneosuppressive cells, including myeloid-derived suppressor cells (MDSCs), cancer-associated fibroblasts (CAFs), M2 subtype of tumor-associated macrophages (M2-TAMs), M1 subtype of tumor-associated macrophages (M1-TAMs), and regulatory T (Treg) cells. A high silencing score was significantly associated with immune inhibition, low expression of LRRC3B, poor patient survival, and activation of cancer-related pathways.

**Conclusion:**

Our comprehensive analysis demonstrated the potential role of LRRC3B in the anti-tumor microenvironment, clinicopathological features of cancer, and disease prognosis. It suggested that LRRC3B methylation could be used as a powerful biomarker to predict immunotherapy responses in NSCLC and BRCA.

## Introduction

The leucine rich repeat containing 3B (LRRC3B) protein has interaction motifs of 20 to 29 amino acid residues characterized by repetition of hydrophobic residues, especially leucine (1–4). LRRC3B acts as tumor suppressor gene, and the expression of LRRC3B in gastric cancer, renal cancer, colorectal cancer (COAD), lung cancer, and breast cancer (BRCA) tissue is lower than in adjacent normal tissue (5). LRRC3B has an essential role in tumorigenesis and cancer progression, and LRRC3B is involved in plant and animal immunity, hormone-receptor interactions, cell adhesion, signal transduction, regulation of gene expression, and apoptosis (6, 7). LRCC3B has also been reported to exert an inhibitory effect on cancer cell colony formation (8). Furthermore, over-expression of LRRC3B was reported to inhibit cell cycle proliferation, invasion, and progression of lung and breast cancer cells, suggesting that LRRC3B may be a useful marker for the diagnosis and prognosis of BRCA and non-small cell lung cancer (NSCLC) (8, 9).

DNA methylation associated with histone modification is a key mechanism to inhibit the expression of tumor suppressor genes in cancer, and DNA methylation markers have been applied in cancer risk assessment, early detection, prognosis, and prediction of response to immune therapy (10–12). For example, methylation of the SHP1 promoter region is involved in tumor immunity and tumor differentiation (13, 14). In addition, TP53 and p16 promoter hypomethylation is significantly inversely associated with gastric cancer risk (15). Interestingly, aberrant DNA methylation of LRRC3B is associated with several types of cancer, including gastric cancer (5), COAD (16), BRCA, lung cancer (17), kidney cancer, ovarian cancer (OV), and prostate cancer (PRCA) (18). More importantly, tumor escape from immune surveillance was reported following silencing of LRRC3B (5). However, no studies have yet systematically explored the protective role of LRRC3B in tumor progression and immunity. Additionally, it is unknown whether LRRC3B DNA methylation levels can serve as a marker to predict response to cancer therapy, especially for anti-PD-1/PD-L1 treatment.

Here, we assess the effects and potential tumor microenvironmental actions of LRRC3B in 33 cancer types from the TCGA databases. We evaluated a number of methylation profiles to assess the effects of LRRC3B silencing on cancer immunotherapy. Our data suggest that LRRC3B is a putative tumor suppressor gene across multiple cancers, including in BRCA, bladder urothelial carcinoma (BLCA), kidney renal clear cell carcinoma (KIRC), skin cutaneous melanoma (SKCM), lung adenocarcinoma (LUAD), and COAD. LRRC3B promoter methylation has significant tumor specificity and may be an early prognostic biomarker to predict immune response in cancers.

## Methods

### mRNA expression analysis and clinical characteristics

**Figure 1:**
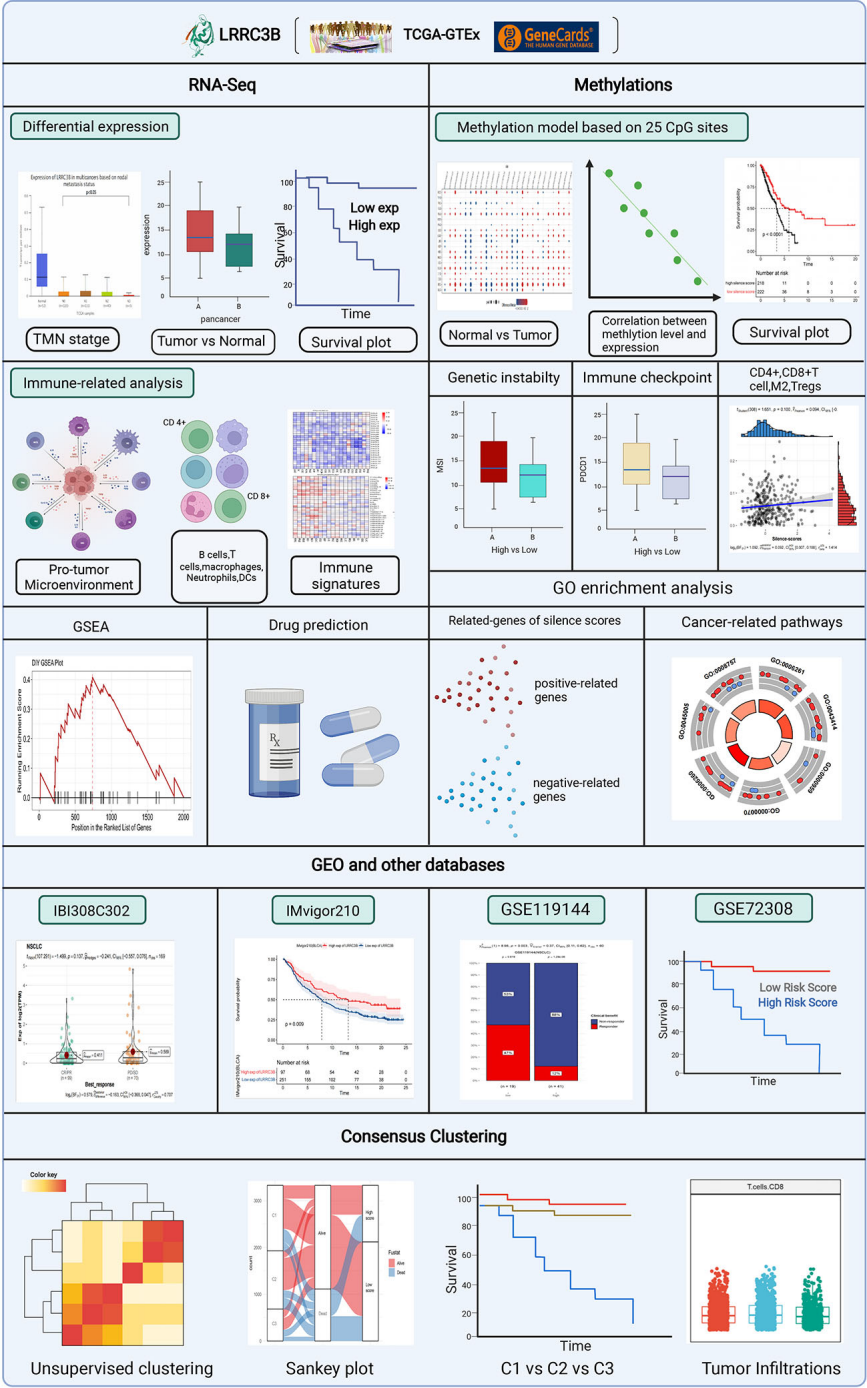
The analytical process to explore the function of LRRC3B in the pan-cancer landscape.

mRNA data from TCGA were obtained using the Xena browser from UCSC (https://xenabrowser.net/datapages/). The logCPM data were used for all mRNA analysis in this study. PROTTER (https://wlab.ethz.ch/protter/start/), an online algorithm, was used to show LRRC3B protein topology, and the expression data for LRRC3B in normal tissue was obtained from Genecard (https://www.genecards.org/). The OPENTARGET platform (https://www.targetvalidation.org/) identifies the involvement of LRRC3B in diseases and aids systematic drug target identification and prioritization. Clinical features of cancer patients were retrieved from Genomic Data Commons. The differential expression analyses of LRRC3B based on clinical stage was performed using the UALCAN website(http://ualcan.path.uab.edu/index.html). A workflow diagram of this study is shown in **Figure 1**.

### Cell lines and culture conditions

BEAS-2B (B2B), H1299, and Hs578T cells were cultured from laboratory stocks. BEAS-2B (B2B) and H1299 cells were cultured in RPMI 1640 medium supplemented with 10% heat-inactivated fetal bovine serum (FBS), 6 mM HEPES, 1.6 mM L-glutamine, 50 μM 2-mercaptoethonal, 100 U/ml Penicillin G, and 100 μg/ml streptomycin sulphate. Hs578T cells were cultured in DMEM medium with 10% FBS and the same supplements. All cell cultures were confirmed to be free of mycoplasma contamination by PCR testing. Authentication testing of BEAS-2B (B2B), H1299, and Hs578T cell lines was accomplished by STR profliling and performed by Shanghai Biowing Applied Biotechnology Co., Ltd.

### qRT-PCR

RNA was extracted from BEAS-2B (B2B), H1299, and Hs578T cells using a TaKaRa MiniBEST Universal RNA Extraction Kit (Code number: 9767). cDNA was generated by using the PrimeScript™ RT reagent Kit (Takara) for RT-PCR. Then, 100 ng RT reaction product was amplified in a 25-AL reaction volume with 2 X SYBR Premix EX Taq (Takara, Code No. RR820A), following the manufacturers protocol, in 96-well format on a CFX96 Real-Time PCR Detection System (Bio-Rad). Primers used were was as follows: LRRC3B forward (5’- TTCCCTCTCCATGTGTCTCC-3’) and reverse (5’-CCAGCATGTTCATCCAACAC-3’). GAPDH forward (5’-GTCTCCTCTGACTTCAACAGCG-3’) and reverse (5’-ACCACCCTGTTGCTGTAGCCAA-3’). GAPDH was used as an internal control. Relative quantification of LRRC3B mRNA was analyzed by the comparative threshold cycle (CT) method (19).

### Immunoblotting

Cells were lysed in RIPA buffer with protease inhibitor cocktail. Total protein was quantified using BCA™ Protein Assay Kit (Thermo). Cell lysates in SDS loading buffer (50 mM TrisHCl pH 6.8, 10% glycerol, 2% SDS, 1% 2-mercaptoethanol, 0.1% bromophenol blue) was boiled and separated by SDS-PAGE (Bio-Rad), then transferred to PVDF membranes (MilliporeSigma). Membranes were immersed in Quickblock blocking buffer (Beyotime Biotechnology, China, cat. no. P0252) for 15 minutes at room temperature before incubation with primary antibodies overnight at 4°C. After washing with TBST, membranes were incubated with the appropriate secondary antibody for 1 h and washed 3 times with TBST. Membranes were incubated in SuperSignal™ West Femto Maximum Sensitivity Substrate (ThermoFisher Scientific) for visualization. Antibodies used were anti-LRRC3B (Affinity Biosciences, DF16048), and anti-vinculin (Cell Signaling, 13901).

### Immunomodulators and immune cells analysis

We conducted Pearson correlation analysis of LRRC3B and immune-related genes, including genes encoding major histocompatibility complex (MHC), immune activation, and chemokines. Six immune cell types (B cells, CD8^+^ T cells, CD4^+^ T cells, macrophages, neutrophils, and dendritic cells) and tumor purity were analyzed using the Tumor IMmune Estimation Resource (TIMER1.0) (Tumor IMmune Estimation Resource (TIMER2.0) (https://cistrome.shinyapps.io/timer/). Myeloid-derived suppressor cells (20, 21) (MDSCs), cancer-associated fibroblasts (CAFs) (22), M2 subtype of tumor-associated macrophages (23) (M2-TAMs), M1 subtype of tumor-associated macrophages (23) (M1-TAMs) and regulatory T (24) (Treg) cells were analyzed using the Sangerbox analysis tool (http://vip.sangerbox.com/).

### Multiplex immunofluorescence staining

We purchased a lung cancer tissue array (HLugA180Su08) from Shanghai Outdo Biotech and performed multiplex immunofluorescence staining (mIHC) using an Opal 7-color fluorescent IHC kit (Catlog Number: NEL801001KT, PerkinElmer) to assess the expression of CD4, CD8, and LRRC3B. Primary antibodies to the following antigens were used: CD4 (ready to use, Abcarta, code number: PA285, monoclonal antibody), CD8 (ready to use, Abcarta, code number: PA067, monoclonal antibody), and LRRC3B (1:200, Affinity Biosciences, code number: DF16048, polyclonal antibody). The primary antibodies were incubated at room temperature for 1h, followed by incubation with secondary antibodies. Nuclei were stained with DAPI. During the process of the mIHC, we first performed pre-experimental optimization of immunohistochemistry and then the polychromatic pre-experiment was performed twice. Fluorescence images were acquired using the TissueFAXS Views software (http://www.jsstl.cn/).

### Genomic instability scores

We curated a list of genomic instability scores, which was composed of the Microsatellite Instability (MSI) score, aneuploidy score, loss of heterozygosity (LOH) score, and the homologous recombination deficiency (HRD) score. The MSI score shows microsatellite loci in tumors appear as novel microsatellite alleles due to the insertion or deletion of repetitive units, compared with normal tissues. The aneuploidy score reports the total number of arm-level amplifications and deletions. The LOH score reports a fraction of genome containing LOH events. The HRD score is combined from the HRD–LOH, LST (large-scale state transitions), and NtAI (number of telomeric allelic imbalances) scores. All scores were evaluated using the Sangerbox analysis tool.

### Genomics of drug sensitivity in cancer and IC50 values

For BRCA, KIRC, LUAD, and SKCM, we used the drugs provided by GDSC (Genomics of Drug Sensitivity in Cancer, GDSC), and calculated the IC50s for different agents using pRRophetic package (25). Spearman correlation analysis evaluating the correlation between LRRC3B expression and IC50 values for different drugs was visualized using the ggplot2 package in R.

### DNA methylation analysis and the LRRC3B silencing score

DNA methylation data from TCGA were collected from the Genomic Data Commons database. We mapped the Illumina methylation array probes to the LRRC3B gene using the Illumina Human Methylation 450 k R annotation data package. All matched probes were retained. We analyzed the differential methylation levels between tumor and normal tissue by Wilcoxon test. According to these probes, we conducted a model of a silencing score which mapped the methylation level of LRRC3B. First, all CpG sites were incorporated into the multivariate Cox regression, and then probes which exhibited a differential methylation at p< 0.05 for each type of cancer were selected to construct the silencing score.


$$silencing\;score=\sum_{i}^{n}\beta_{i}*M_{i}$$

(*β_i_* represents the coefficient index, and *M_i_* represents the methylation level)

To examine the regulation of LRRC3B expression by DNA methylation level, we calculated the Spearman correlation between the silencing score with mRNA expression for LRRC3B.

### Survival analysis

Survival analyses of gene expression from RNA-sequencing among multiple cancers was performed using the KM-Plotter tool. In brief, Kaplan-Meier survival curves for overall survival (OS) were generated for each cancer type using the Survival and Survminer R packages to test the association between the silencing score and survival.

The optimal cut-off values for each cancer were determined based on X-tile. Subsequently, patients were divided into high- and low-risk groups based on the silencing score according to the optimal cut off value. A statistically significant difference in survival was denoted by a log-rank test p-value of p< 0.05. Cancers were divided into three clusters based on consensus clustering of DNA methylation analysis of LRRC3B. Survival analysis between the three clusters was performed using the log-rank test.

### Immune signature of the silencing score and copy number alteration

We used the CIBERSORTx algorithm to estimate the proportions of immune cells. Pearson correlation analysis for CD4^+^T cells, CD8^+^T cells, Tregs, M2-TAMs, was conducted among nine different cancers (BRCA, BLCA, COAD, GBM, KIRC, LUAD, LUSC, SARC, SKCM). The differences between copy number alteration (CNA) of LRRC3B and immune infiltration were analyzed using theTumor IMmune Estimation Resource (TIMER 2.0).

### Biological pathway analysis

We identified biological pathways of hallmark gene sets associated with the expression of LRRC3B using the GSEA Java (v4.1.0). We reported pathways showing negative or positive correlation with LRRC3B in 12 cancer types. Regarding the silencing score, 300 genes with top positive and negative pearson correlation to the silencing score were selected to perform Gene Ontology (GO) enrichment analysis using the “clusterProfile” R package, and were visualized by the “GOplot” R package.

### Prediction of response to immunotherapy cohort

Gene expression data for immunotherapy response and overall survival data were obtained from the IMvigor210 public cohort (patients with advanced or metastatic urothelial carcinoma treated with atezolizumab therapy). DNA methylation data from the GSE119144 (26) and GSE72308 (27)cohorts was used to predict response to anti-PD-1 treatment. Generation of Kaplan-Meier survival curves of overall survival was performed using the Survival and Survminer R packages, and the log-rank test was used to test the statistical significance of the difference in survival between the low and high LRRC3B methylation groups. The proportional difference of clinical benefits in GSE119144 between the low and high groups was analyzed by the Chi-Squared Test, and a p-value of p< 0.05 indicated a statistically significant difference.

## Results

### LRRC3B localization and normal tissue expression profile

**Figure 2:**
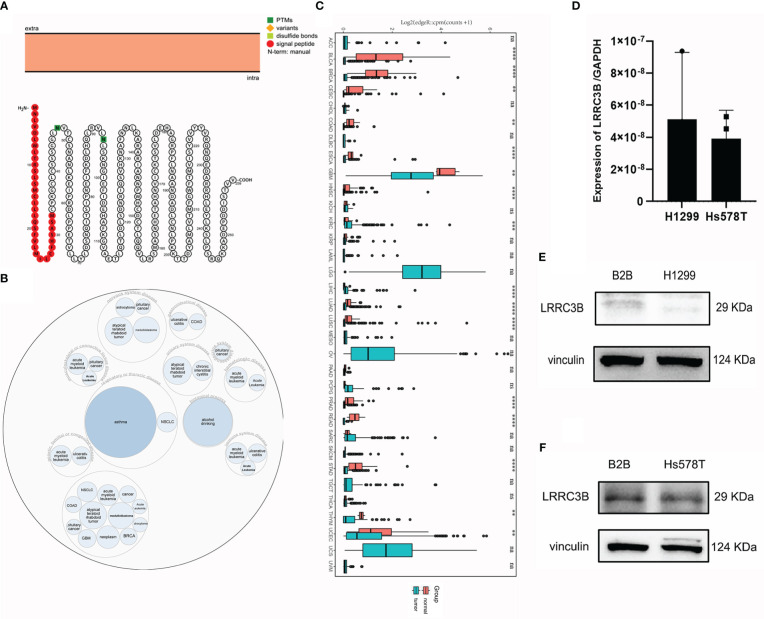
LRRC3B variants, localization, and expression profile under physiological conditions. **(A)** The LRRC3B protein topology revealed extracellular membrane localization with PTMs. **(B)** The LRRC3B-associated disease network. **(C)** Boxplots showing differential LRRC3B expression levels (log CPM+1) between tumor and adjacent normal tissues across the TCGA database. **(D)** Real-time RT-PCR analysis of LRRC3B mRNA in H1299 and Hs578T cell lines. **(E)** Protein levels of LRRC3B in B2B and H1299 cell lines assessed by immunoblot. **(F)** Protein levels of LRRC3B in B2B and Hs578T cell lines assessed by immunoblot. LRRC3B: leucine rich repeat containing 3B; PTMs: protein post-translational modifications; TCGA: The Cancer Genome Atlas.

We outline the entire process of the study in **Figure 1**, including bulk RNA-sequencing and DNA methylation data analysis. First, we explore a tumor suppressor role for LRRC3B in different cancer types by differential analysis, immune cell infiltration, and predictive power of relative drugs. Second, DNA methylation of LRRC3B was used to construct a silencing score. Finally, public datasets including data from patients treated with immunotherapy were utilized to validate the results. The LRRC3B protein topology revealed extracellular membrane localization with protein post-translational modifications (PTMs), showing N-methylation, which can be involved in cell differentiation, protein degradation, signal transduction and regulation, gene expression regulation, and protein interaction (**Figure 2A**). A gene and disease network interaction analysis revealed that LRRC3B was associated with metabolic diseases, cell proliferation, immune system, and multiple cancers (**Figure 2B**). Furthermore, through analyzing data from TCGA, we found that LRRC3B mRNA expression is significantly lower in tumors of multiple cancer types compared with their corresponding adjacent normal tissues (**Figure 2C**). LRRC3B levels were measured by qPCR in H1299 (lung cancer cell) and Hs578T (breast cancer cell) cells, but were very low and barely detected (**Figure 2D**). The difference of LRRC3B protein expression between normal lung epithelial cells and cancer cells was measured by immunoblotting (**Figures 2E, F**). Compared with B2B cells, H1299 and Hs578T cells showed lower expression, though significant difference in LRRC3B expression was detected between Hs578T and B2B cells.

### Higher LRRC3B is associated with lower TMN stage and better cancer prognoses

**Figure 3:**
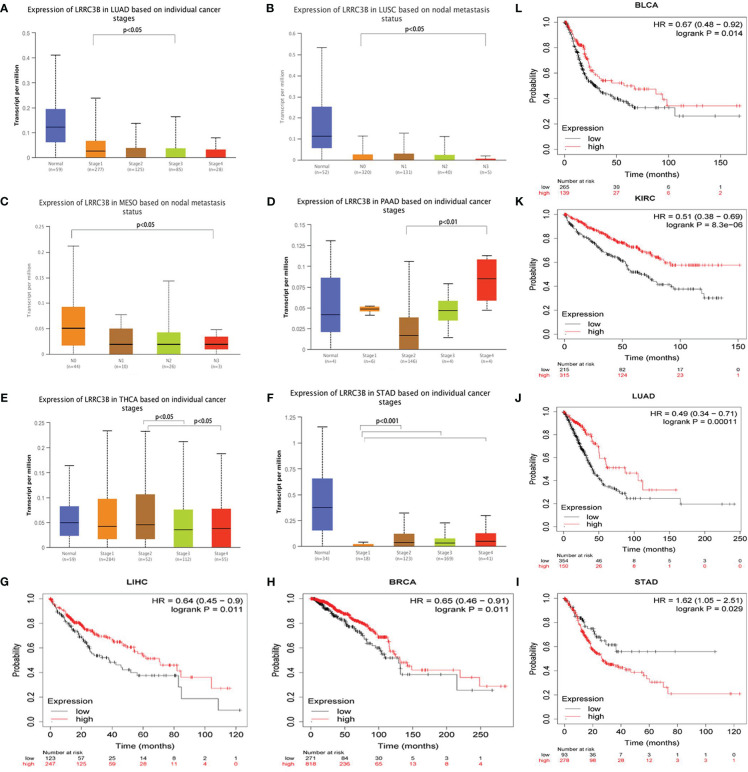
The clinical features of LRRC3B in different cancers. **(A-F)** Boxplot showing differential LRRC3B expression levels between pathological stages (stages I, II, III, and IV), and lymph node status. **(G-L)** Kaplan-Meier curves of overall survival differences based on high and low expression levels of LRRC3B in TCGA cancer cohorts. Only TCGA cancers with statistically significant differences are presented. LRRC3B: leucine rich repeat containing 3B; TCGA: The Cancer Genome Atlas.

To further explore the role of LRRC3B in tumor progression, we compared the expression levels of LRRC3B based on tumor stage. We found that LRRC3B expression was lower at higher tumor stages in LUAD, MESO, LUSC, and THCA (**Figures 3A, B, C, E**), and was increased in STAD and PAAD (**Figures 3D, F**). Consistently, overexpression of LRRC3B was associated with better OS of patients with BLCA (p = 0.014), BRCA (p = 0.011), LUAD (p< 0.001), KIRC (p< 0.0001), and LICH (p = 0.011) (**Figures 3G, H, J, K, L**). However, overexpression of LRRC3B was associated with poorer survival in STAD (p = 0.029). Altogether, these findings strongly suggest that LRRC3B has tumor suppressor qualities and is associated with slower tumor progression, better tumor staging, and reduced metastasis, and could serve as early biomarker for cancer detection, staging, and follow-up.

### LRRC3B expression-associated immune signatures, genomic instability, and biological pathways

**Figure 4:**
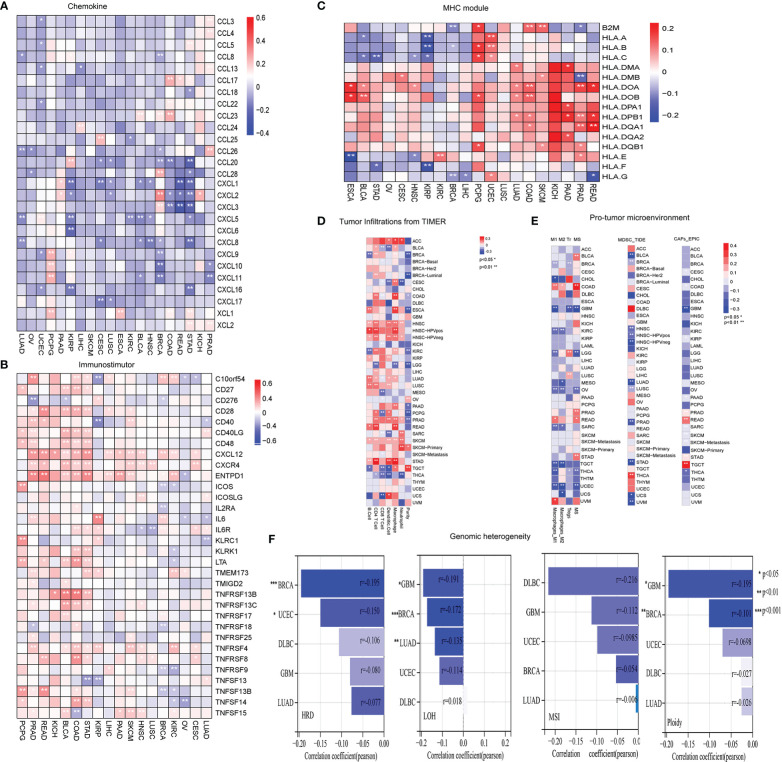
Evaluation of tumor microenvironment and genomic instability in different cancers. **(A)** Heatmap showing negative correlations of LRRC3B expression with chemokine genes. **(B)** Heatmap showing positive correlations of LRRC3B expression with immunostimulatory genes. **(C)** Heatmap showing positive correlations of LRRC3B expression with MHC molecules. **(D)** Pearson correlation of LRRC3B expression with six immune cell types and tumor purity. **(E)** Pearson correlation analysis of LRRC3B expression with myeloid-derived suppressor cells, cancer-associated fibroblasts, M2 subtype of tumor-associated macrophages, M1 subtype of tumor-associated macrophages, and regulatory T cells. **(F)** Correlations of LRRC3B expression with genomic instability scores. LRRC3B: leucine rich repeat containing 3B; CAFs: cancer-associated fibroblasts; MDSCs: myeloid-derived suppressor cells; Tr: regulatory T cell; M2: M2 subtype of tumor-associated macrophages; M1: M1 subtype of tumor-associated macrophages; MSI:microsatellite Instability; ploidy: aneuploidy; LOH: loss of heterozygosity; HRD:homologous recombination deficiency.

To better understand the molecular relationship between expression of LRRC3B and tumor immunity, we calculated the Pearson correlation of LRRC3B expression with a panel of immunomodulators, including chemokines, immunostimulators, and MHC modules, that are crucial in immunotherapy (28). LRRC3B expression was negatively correlated with most chemokines (CXCL1/2/3/5/6/8/9/10, CCL20, and CCL28) in more than 18 cancers, positively correlated with immunostimulators (CD27, CD28, CD40, TNFRSF13B, TNFRSF8/9, and TNFSF13) and MHC I/II modules, especially in KICH, COAD, BLCA, PRAD, PCPG, READ, and SCKM (**Figures 4A-C**).

**Figure 5:**
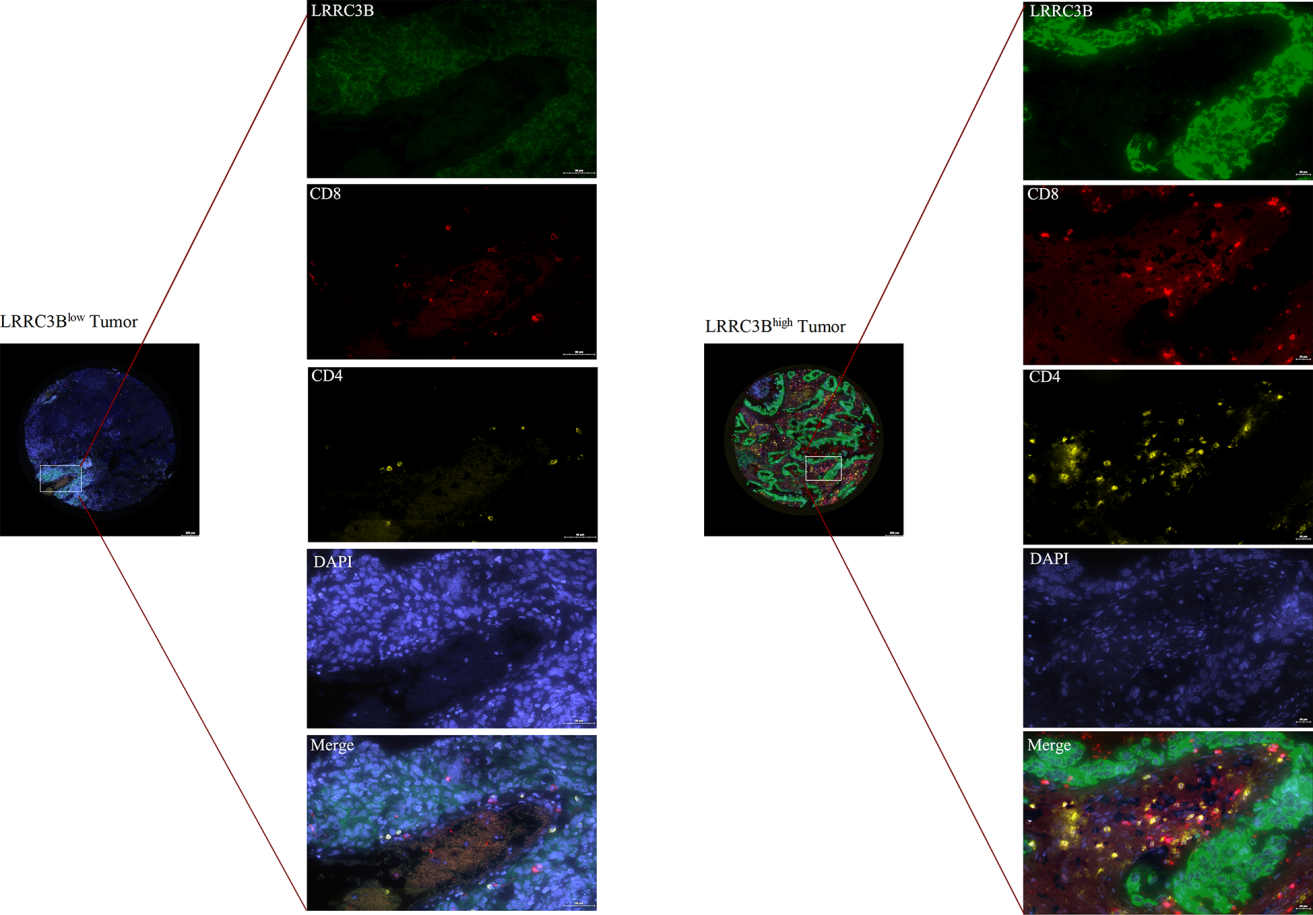
Multiplex IHC images of lung cancer tissues with immune cell biomarkers and LRRC3B expression. mIHC: multiplex immunofluorescence staining; LRRC3B: leucine rich repeat containing 3B.

We next examined the relationship between LRRC3B expression levels and the levels of infiltration of 11 immune-related cells. Consistent with the results from our survival analysis, overexpression of LRRC3B was associated with infiltration of anti-tumor immune cells (**Figure 4D**), including B cells, CD4^+^ T cells, CD8^+^ T cells and antigen presentation cell, while it suppressed the levels of infiltrating M2 macrophages, MDSCs, CAFs, and Tregs (**Figure 4E**). Additionally, we found that LRRC3B expression was associated with lower levels of MSI, HRD, LOH and aneuploidy scores for genome instability, which revealed that LRRC3B may function to maintain genomic integrity in BRCA, UCEC, DLBC, GBM, and LUAD (**Figure 4F**). We performed authentication testing of the correlation between LRRC3B expression and infiltration of CD4^+^/CD8^+^ T cell, using multiplexed fluorescence immunohistochemistry (mIHC), in the lung cancer tissue array. We observed a higher degree of infiltration of CD4^+^/CD8^+^ T cells in tumors with high-expression of LRRC3B (**Figure 5**), which was consistent with our bioinformatics analysis. This provides strong evidence that LRRC3B might play an essential role in tumor inhibition facilitated through CD4^+^/CD8^+^ T cells.

**Figure 6:**
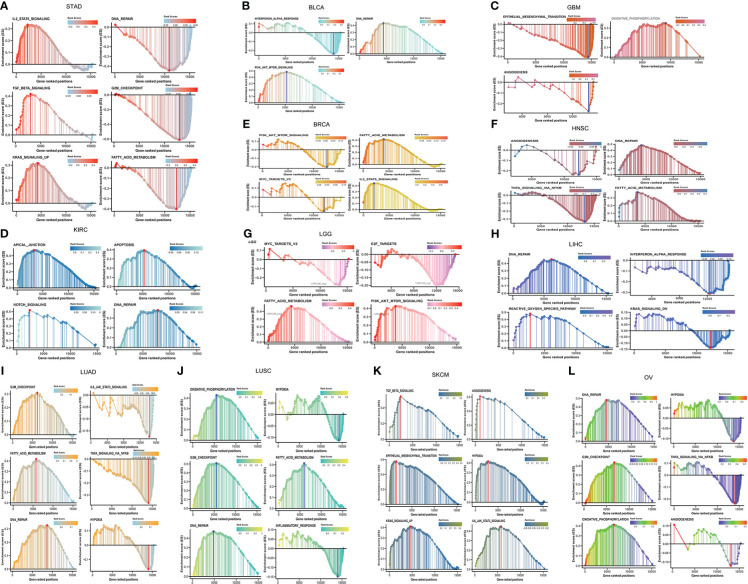
Gene set enrichment and pathways associated with LRRC3B expression in **(A)** STAD, **(B)** BLCA, **(C)** GBM, **(D)** KIRC, **(E)** BRCA, **(F)** HNSC, **(G)** LGG, **(H)** LIHC, **(I)** LUAD, **(J)** LUSC, **(K)** SKCM, **(L)** OV.

LRRC3B has many biological functions, such as regulation of cell proliferation, cell cycle progression, and tumor escape from immune surveillance. To explore pathways associated with LRRC3B expression, we performed Pearson correlation analysis for LRRC3B expression and hallmark gene sets by GSEA analysis. GSEA analysis revealed pathways both positively and negatively correlated with LRRC3B expression, in a context dependent setting. Tumor suppressor pathways, such as fatty acid metabolism, DNA repair, oxidative phosphorylation, and G2M checkpoint, were found to positively associate with LRRC3B expression in 12 cancer types (LUAD, LUSC, BRCA, BLCA, KIRC, HNSC, OV, STAD, LGG, GBM, SKCM, LIHC), suggesting that LRRC3B is involved in regulating cell metabolism and cell cycle progress (**Figure 6**). TNF-alpha signaling *via* NF-kB, IL6/JAK/STAT3 signaling, PI3K/AKT/mTOR signaling, MYC targets v2, TGF-beta signaling, and Kras signaling were mostly negatively correlated with LRRC3B expression, consistent with silencing of LRRC3B in tumor tissue, which may result in carcinogenesis.

### LRRC3B expression predicts drug sensitivity to small molecule inhibitors

**Figure 7:**
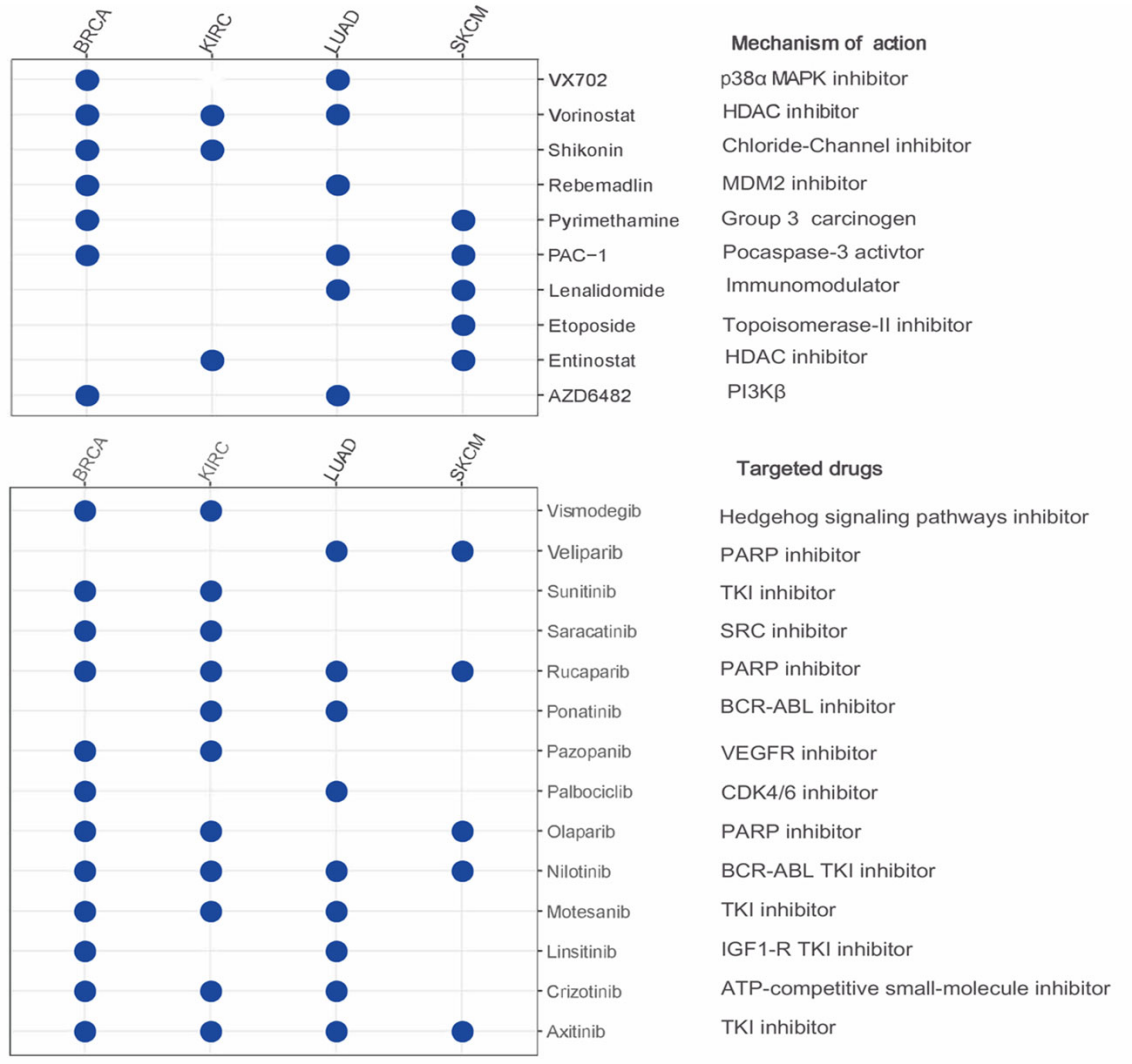
The relationship between LRRC3B and anti-tumor drugs. Negative correlation of LRRC3B with IC50 value of small molecule inhibitors. LRRC3B: leucine rich repeat containing 3B.

We next employed the pRRophetic method by calculating the IC50 of a variety of drugs to search for candidate compounds that might modulate LRRC3B expression. Drugs including AZD6482 (PI3Kβ inhibitor), VX702 (p38α MAPK inhibitor) and targeted drugs (such as nilotinib, olaparib, sunitinib, crizotinib) had IC50 values that were negatively correlated with LRRC3B expression (**Figure 7**), illustrating that overexpression LRRC3B may enhance sensitivity to certain agents.

### LRRC38 silencing score predicts patient survival and may indicate tumor escape from immune surveillance

**Figure 8:**
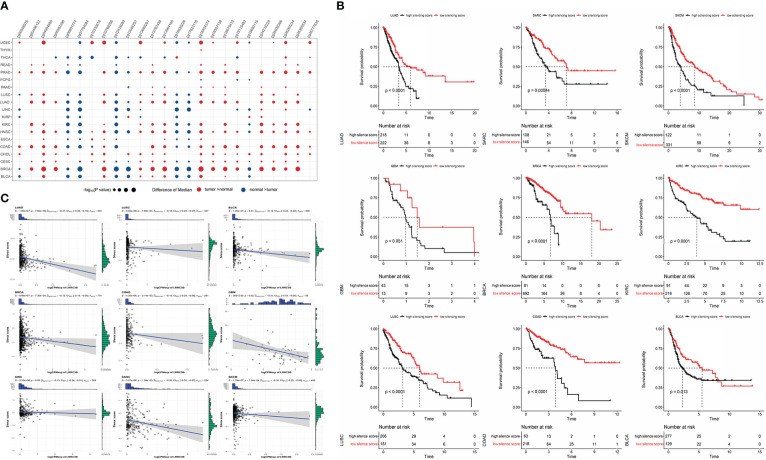
Construction of silencing score based on 25 CpG sites of LRRC3B. **(A)** Dotplots showing differential LRRC3B methylation levels between tumor and adjacent normal tissues across TCGA database by nonparametric testing. **(B)** Kaplan–Meier analysis of OS between the high and low silencing score groups. **(C)** The silencing score was negatively correlated with LRRC3B mRNA expression. LRRC3B, leucine rich repeat containing 3B; OS, overall survival; TCGA, The Cancer Genome Atlas.

We found that the levels of DNA methylation of ~25 CpG sites were significantly higher in tumor tissues compared to adjacent normal tissues in most TCGA cancers (**Figure 8A**).

Therefore, we computed a “silencing score”, which represents an estimated methylation level of LRRC3B. Given the important association of DNA methylation with gene expression, we evaluated whether this silencing score was predictive of patient survival. Patients were divided into two groups based on the silencing score and we performed Kaplan-Meier survival curve analysis between the high- and the low-silencing score groups. Among 33 cancer types, we found that low silencing score was associated with better survival in BRCA, BLCA, COAD, KIRC, LUAD, LUSC, SKCM, GBM, and SARC (**Figure 8B**).

In a study by Saghafinia S., et al. (29), DNA hypermethylation at the promoter region was shown to inhibit the expression of the downstream gene, demonstrating that DNA methylation represents a key regulatory element of gene expression. In these nine cancers, Spearman correlation analysis showed that the silencing score was negatively correlated with LRRC3B mRNA expression (**Figure 8C**), fully consistent with the inferred inhibition of LRRC3B suggested by the DNA methylation levels.

**Figure 9:**
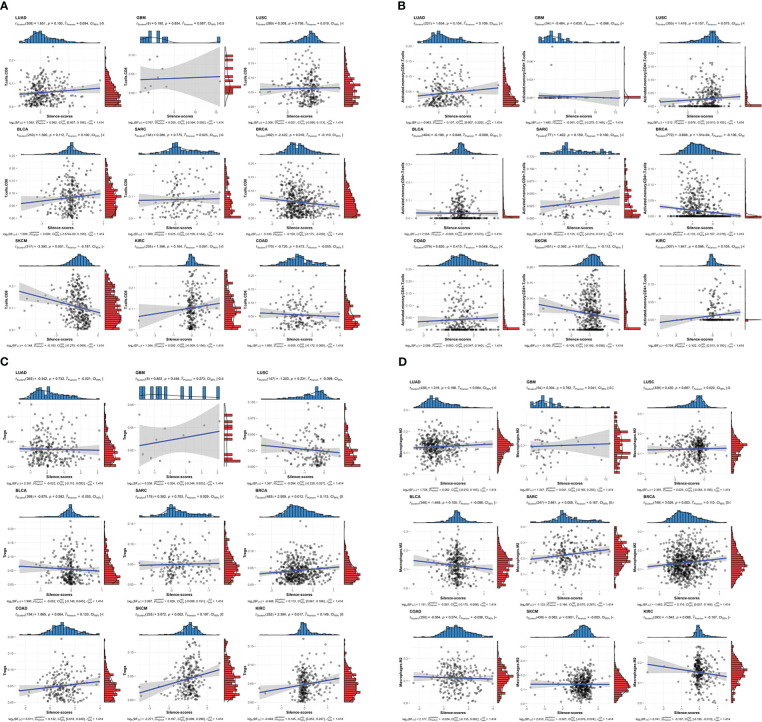
Scatterplots of correlations of silencing scores with immune infiltration. **(A)** CD8+ T cell, **(B)** activated memory CD4^+^ T cell, **(C)** Tregs, and **(D)** M2 macrophages. Tregs, regulatory T cells.

**Figure 10:**
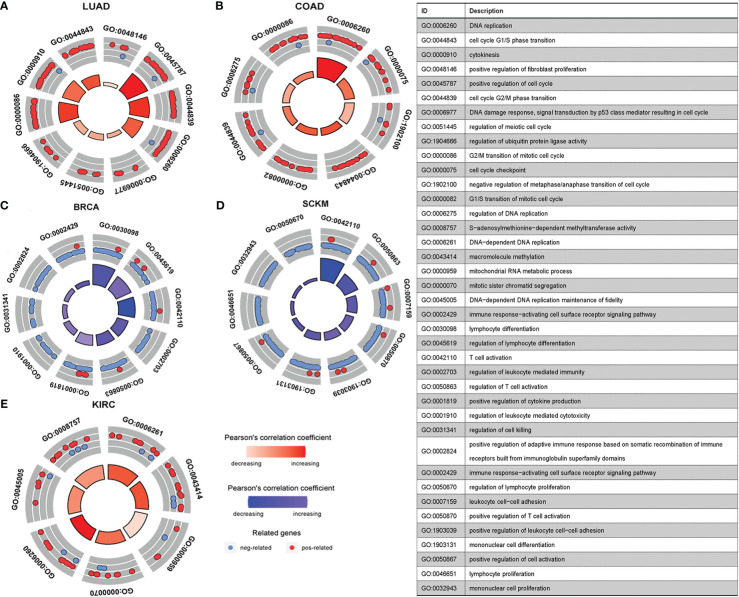
GO enrichment pathways of silencing scores. **(A, B, E)** Pathways positively correlated with silencing score. **(C, D)** Pathways negatively correlated with silencing score. GO, Gene Ontology.

Having established the link between silencing score and LRRC3B expression, we explored immunity based on methylation levels to further show that LRRC3B plays a crucial role in the tumor microenvironment. Nine tumors, including BRCA, BLCA, COAD, KIRC, LUAD, LUSC, SKCM, GBM, and SARC, with the Pearson correlation between silencing score and immune cell infiltration, were screened for further analyses (**Figure 9**). Silencing score was negatively correlated with levels of activated memory CD4^+^ T cells and CD8^+^ T cells in BRCA and SKCM, and was positively associated with levels of infiltrating M2 macrophages and Tregs in BRCA, SKCM, and SARC. According to the biological pathways associated with the silencing score, GO enrichment analyses (**Figure 10**) demonstrated that the silencing score exerted a positive influence on DNA damage, cell proliferation, and cell cycle progression, such as G2/M transition, DNA replication, and positive regulation of fibroblast proliferation. Conversely, the silencing score exerted a negative influence on T cell activation, regulation of cell killing, and regulation of leukocyte mediated cytotoxicity.

Altogether, we conclude that LRRC3B silencing by DNA methylation at the promoter region induces tumor escape from immune surveillance by decreasing levels of anti-tumor cells in the TME, inhibiting regulation of cell killing, increasing the levels of pro-tumor cells in the TME, and promoting cancer cell proliferation.

**Figure 11:**
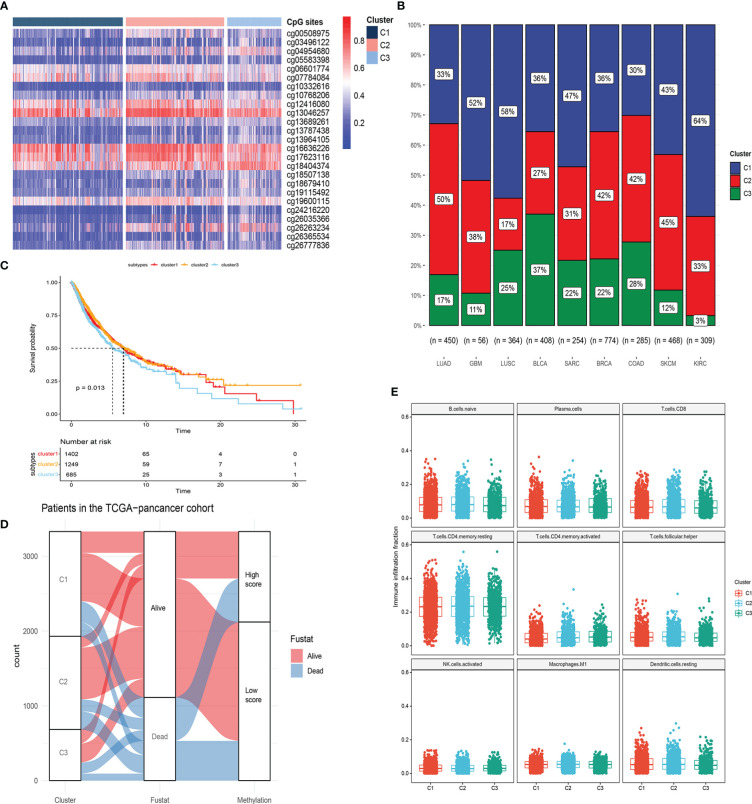
Molecular subtypes based on 25 CpG sites within LRRC3B locus by unsupervised consensus clustering. **(A, B)** Unsupervised clustering of DNA methylation and sample distribution in the three clusters. **(C)** Survival analysis of three subtypes. **(D)** Alluvial diagram of subtype distributions in groups with different silencing scores and survival outcomes. **(E)** Differences in immune infiltration scores among the three gene subtypes.

To obtain a comprehensive picture of the DNA methylation levels of LRRC3B, we next performed an unsupervised consensus clustering of all 25 CpG sites in the nine cancers that were related to patients’ survival. Three subtypes of samples emerged (C1; n =1402, C2; n =1249, C3; n =685), with C3 displaying hypomethylation compared with C2 and C1 (**Figure 11A**). Sample distribution in the three clusters is shown in **Figure 11B**. We also examined the overall survival rate of the three groups, and found that patients in C1 and C2 displayed better survival than in C3 (**Figures 11C**, p = 0.013). From the Sankey plot, patients who lived mostly belonged to the C1 and C2 subtypes, corresponding to low silencing score group (**Figure 11D**). Our immune analysis (**Figure 11E**) indicated that C1 and C2 showed a significantly higher enrichment than C3 in immune cells, thus also identifying the close relationship methylation subtypes and silencing score. In total, the DNA hypomethylation of LRRC3B could predict better OS.

### Epigenetic prediction of response to anti-PD-1 treatment

**Figure 12:**
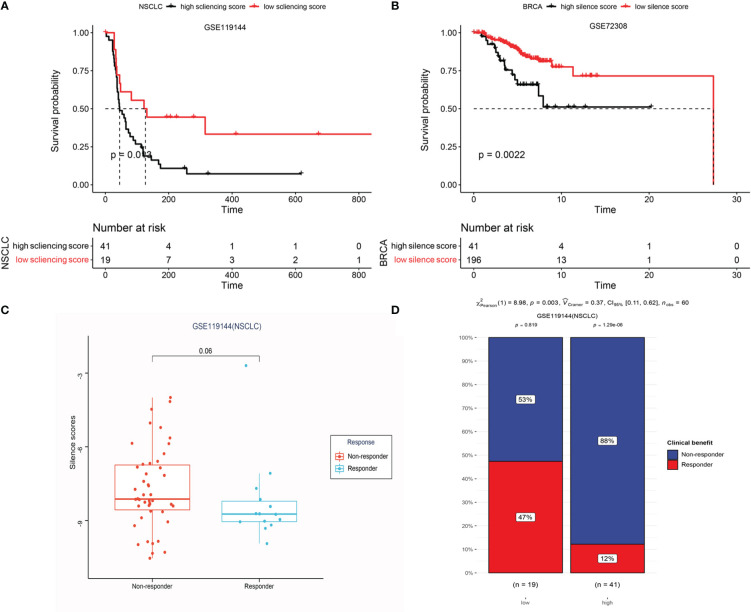
Epigenetic prediction of response to anti-PD-1 treatment. **(A)** Kaplan-Meier curves display the overall survival of patients in the high and low-risk group in GSE119144. **(B)** LRRC3B promoter hypermethylation is associated with unfavorable OS in BRCA (GSE72308). **(C)** Differential silencing score between non-responders and responders. **(D)** Exploring the risk factor (different risk groups) for immunotherapy response in GSE119144 cohorts (Chi-square test).

To define an epigenomic profile associated with patients who would gain clinical benefit from anti-PD-1 treatment, we first plotted box plots of high and low silencing score group differences in immune checkpoints, like PDCD1,CTLA4, and CD274, crucial factors in immunotherapy. We noted significant inter-group differences (**Figure S1**). Notably, the box plots revealed that low silencing score showed a significantly higher expression of checkpoints in BRCA, LUAD, LIRC, and SKCM, possibly displaying a better clinical benefit from immunotherapy. To further validate these results, anti-PD-1 treatment cohorts based on methylation profiling (GSE119144 and GSE72308) from the GEO database were analyzed. We found that low silencing score, representing DNA hypomethylation, was significantly associated with improved progression free survival and overall survival in patients with NSCLC and BRCA, respectively (**Figures 12A,B**). Furthermore, responders to anti-PD1 immunotherapy showed lower methylation levels (**Figure 12C**), and in different silencing score groups (high vs low), the patients exhibited significant differences in immune response (Non-response vs. response) (**Figures 12D**, p = 0.003). These data suggest that DNA hypomethylation of LRRC3B could be used to predict anti-PD-1 treatment outcomes in NSCLC and BRCA.

### Additional analyses of genomic instability scores and copy number alterations

In a study by Jung, Hyunchul et al., genomic hypomethylation was shown to be assioated with immune escape signatures of aneuploid tumors (26). Hence, we compared the genomic instability scores (MSI, LOH, HRD, and aneuploidy score) between the high- and low- silencing score groups. A low silencing score was associated with lower genomic instability, especially in BRCA, LUAD, and SARC (**Figure S2**). To complete a comprehensive analysis of LRRC3B, we further evaluated the differences of tumor immune infiltration across deep deletion, arm-level deletion, arm-level gain, and high amplification, compared with normal tissue (**Figure S3**). We found that copy number variation of LRRC3B decreased the infiltration of immune cells and possibly promoted tumor progression.

## Discussion

LRRC3B encodes a leucine-rich repeat protein that inhibits tumor proliferation, colony formation, and invasion (30). As a tumor suppressor gene, LRRC3B exerts tumor suppressor activity through immune responses. Mediators of immune response, cell adhesion, cell growth, cell death, intercellular signaling, and metabolism were all up-regulated in LRRC3B-expressing xenograft tumor models, and showed higher immune scores of activated CD4 T cells, and activated CD8

T cells. Thus, we explored the tumor suppressor role of LRRC3B in multiple cancers. Our results suggest that the expression of LRRC3B was repressed in most of TCGA cancers, especially at higher tumor stages. Moreover, overexpression of LRRC3B was associated with better survival in several cancers, such as BLCA, BRCA, LUAD, KIRC, and LICH. Overexpression of LRRC3B was found to associate with increased abundance of B cells, CD4^+^ T cells, CD8^+^ T cells, and antigen presentation cells, and suppressed the infiltration of M2 macrophages, MDSCs, CAFs, and Treg cells. MDSCs are reported to exert immunosuppressive functions in tumor immunotherapy, and the overexpression of LRRC3B may a potential approach to enhance anti-PD-L1 cancer immunotherapy (31–33), although this requires further validation.

MHC class I antigens are important in recognition by the immune system. Down-regulation of MHC class I antigens and loss of tumor antigens contribute to tumor cell escape from immune surveillance (34). We also found that levels of immunostimulators (CD28, CD40, CD27) and MHC molecules were positively correlated with LRRC3B expression (**Figures 4B, C**).

High LRRC3B expression predicted better clinical outcomes in an immunotherapy cohort. Targeting major tumor suppressors could help tumor cells escape growth control by molecular signaling pathways such as p53 signaling (35). Tumor necrosis factor alpha (TNF-α) is thought to be a vital factor promoting tumor immunosuppression by escaping T cell surveillance and stabilizing programmed cell death-ligand 1 (36). In addition, tumor suppressor genes (BRCA1 and BRCA2) are involved in DNA repair and transcriptional regulation in response to DNA damage, thereby suppressing tumorigenesis (37). Our bioinformatics analysis showed that DNA repair, oxidative phosphorylation, and G2M checkpoint were positively associated with LRRC3B expression in a number of cancers: LUAD, LUSC, BRCA, BLCA, KIRC, HNSC, OV, STAD, LGG, GBM, SKCM, and LIHC. Conversely, TNF-alpha signaling *via* NF-kB, IL6/JAK/STAT3 signaling, PI3K/AKT/mTOR signaling, MYC targets v2, TGF-beta signaling, and KRAS signaling were mostly negatively correlated with LRRC3B expression. These analyses further illustrate the anti-tumor effects of LRRC3B in multiple cancers.

Silencing by promoter hypermethylation was the main mechanism of LRRC3B inactivation, and it was reported that six CpG sites around the first CpG island (chr3:26664104-26664796) in the promoter region of LRRC3B gene show hypermethylation when compared with normal tissues (38). Consistently, our results also showed that the methylation level of LRRC3B is significantly higher in cancer tissues than in the normal adjacent tissues. LRRC3B DNA methylation levels may be a marker that can be used for cancer diagnosis and prognosis (8, 16, 39). We constructed a model of a silencing score based on the methylation level of LRRC3B, and estimated the effects of this silencing score to predict patient survival. Among 33 cancer types, we found that low methylation of LRRC3B was associated with better survival in BRCA, BLCA, COAD, KIRC, LUAD, LUSC, SKCM, GBM, and SARC. We also found that expression of LRRC3B negatively correlated with the silencing score. Epigenetic silencing in tumors is a major factor associated with tumor progression (11). Our results show that lower expression levels of LRRC3B are likely attributed to DNA hypermethylation of LRRC3B at the promoter region.

Studies have shown that tumor-associated macrophages and neutrophils, cancer-associated fibroblasts, and senescent endothelial cells are inversely associated with DNA methylation signatures, as assessed by IHC (40, 41). Moreover, tumors with “immune cold” microenvironments may be ineffective targets for anti-PD-1 therapy (40), mainly due to weakened immune surveillance that is unable to suppress tumor cell growth (42). Monoclonal antibodies widely used in immune control, such as PD-1, PD-L1, and cytotoxic T lymphocyte antigen 4 (CTLA-4), have significantly improved the prognosis of patients with advanced cancer (43). Interestingly, CTLA4 promoter methylation appears to be able to predict immune checkpoint blockade responses in SKCM and KIRC (44). In our study, we found that DNA hypomethylation of LRRC3B could be used to predict anti-PD-1 treatment outcomes in NSCLC and BRCA. Analysis of immune infiltration indicated that the abundance of activated memory CD4^+^ T cells and CD8^+^ T cells are associated with a DNA methylation-negative signature in BRCA and SKCM, while immune checkpoints, such as PDCD1, CTLA4, and CD274 displayed higher expression in the low silencing score group. These data suggest that hypermethylation of LRRC3B may induce tumor immune escape. More importantly, genomic instability facilitates cytotoxin escape (41). We found that genomic instability scores were correlated with hypermethylation of LRRC3B, which suggested that hypomethylation of LRRC3B in BRCA and NSCLC could prevent tumor escape from immune surveillance. These data indicate that demethylating drugs may be able to convert an immune cold tumor to an immune active tumor, and may combine with immunotherapy to achieve better clinical benefits (45).

In conclusion, we report that LRRC3B may exert anti-tumor effects in multiple cancer types, and may be an essential actor in the tumor microenvironment to determine the response to immune checkpoint inhibitors. We established a DNA methylation model of LRRC3B in NSCLC and BRCA to be a predictive tool for selecting patients who may achieve better clinical benefit from anti-PD-1 therapy. Our findings also provided evidence that hypermethylation of the LRRC3B promoter may facilitate tumor escape from immune surveillance. In the future, a prospective clinical studies would be warranted to verify the value of the prognostic and predictive benefit of the LRRC3B DNA methylation model.

## Data availability statement

Publicly available datasets were analyzed in this study. This data can be found here: https://xenabrowser.net/datapages/, and GEO(https://www.ncbi.nlm.nih.gov/geo/query/acc.cgi?acc=GSE119144/GSE72308). Gene expression data for immunotherapy response and overall survival data were obtained from the IMvigor210 public cohort.

## Author contributions

LL and SH designed the study. LL and SF analyzed the data and designed the figures. WD and L-nH revised the manuscript. XZ, YW, and YZ collected the clinical information of the immunotherapy cohorts. All authors contributed to the article and approved the submitted version.

## Glossary

**Table:**

LRRC3B	Leucine rich repeat containing 3B
ACC	adrenocortical carcinoma
BLCA	bladder urothelial carcinoma
BRCA	breast invasive carcinoma;
CESC	cervical squamous cell carcinoma and endocervical adenocarcinoma
CHOL	cholangiocarcinoma
COAD	colon adenocarcinoma
DLBC	lymphoid neoplasm diffuse large B-cell lymphoma
ESCA	esophageal carcinoma
GBM	glioblastoma;
HNSC	head and neck squamous cell carcinoma
KICH	kidney chromophobe;
KIRC	kidney renal clear cell carcinoma
KIRP	kidney renal papillary cell carcinoma
LAML	acute myeloid leukemia
LGG	brain lower grade glioma;
LIHC	liver hepatocellular carcinoma
LUAD	lung adenocarcinoma
LUSC	lung squamous cell carcinoma
MESO	mesothelioma
OV	ovarian serous cystadenocarcinoma
PAAD	pancreatic adenocarcinoma
PCPG	pheochromocytoma and paraganglioma
PRAD	prostate adenocarcinoma;
READ	pectum adenocarcinoma
SARC	sarcoma
SKCM	skin cutaneous melanoma
STAD	stomach adenocarcinoma
TGCT	testicular germ cell tumors;
THCA	thyroid carcinoma
THYM	thymoma
UCEC	uterine corpus endometrial carcinoma
UCS	uterine carcinosarcoma
UV	uveal melanoma
NSCLC	non-small cell lung cancer
MHC	major histocompatibility complex
MDSCs	myeloidderived suppressor cells
CAFs	cancer-associated fibroblasts
M2-TAMs	M2 subtype of tumor-associated macrophages
M1-TAMs	M1 subtype of tumorassociated macrophages
Treg	regulatory T
MSI	microsatellite instability
LOH	loss of heterozyosity
HRD	homologous recombination deficiency
LST	large-scale state transitions
NtAI	number of telomeric allelic imbalances
CNA	copy number alteration
GO	gene ontology
PTMs	protein post-translational modifications;
IHC	immunohistochemistry
mIHC	multiplex immunofluorescence staining;
CTLA-4	cytotoxic T lymphocyte antigen 4.
