# Supplementary material for: LRRC3B and its promoter hypomethylation status predicts response to anti-PD-1 based immunotherapy
Source: Front Immunol. 2023 Jan 24;14:959868. doi: 10.3389/fimmu.2023.959868 (PMC9928207; doi:10.3389/fimmu.2023.959868)
Supplement: Supplementary file 1 [file DataSheet_1.docx]

Supplementary Material

Supplementary Tables

**Table S1.** Pearson’s correlation between LRRC3B expression and pro-tumor immune cell infiltration scores .

**Table S2.** Pearson’s correlation between LRRC3B expression and anti-tumor immune cell infiltration scores .

**Table S3.**Pearson’s correlation between LRRC3B expression and a panel of immunomodulators

**Table S4.** Pearson’s correlation between LRRC3B expression and Genomic instability scores.

**Table S5.** Pearson correlation analysis for LRRC3B expression and relative biological pathways of hallmark gene sets by GSEA analysis.

**Table S6.** The IC50 value by using pRRophetic package.

**Table S7.** GO pathways based on DNA methylation of LRRC3B.


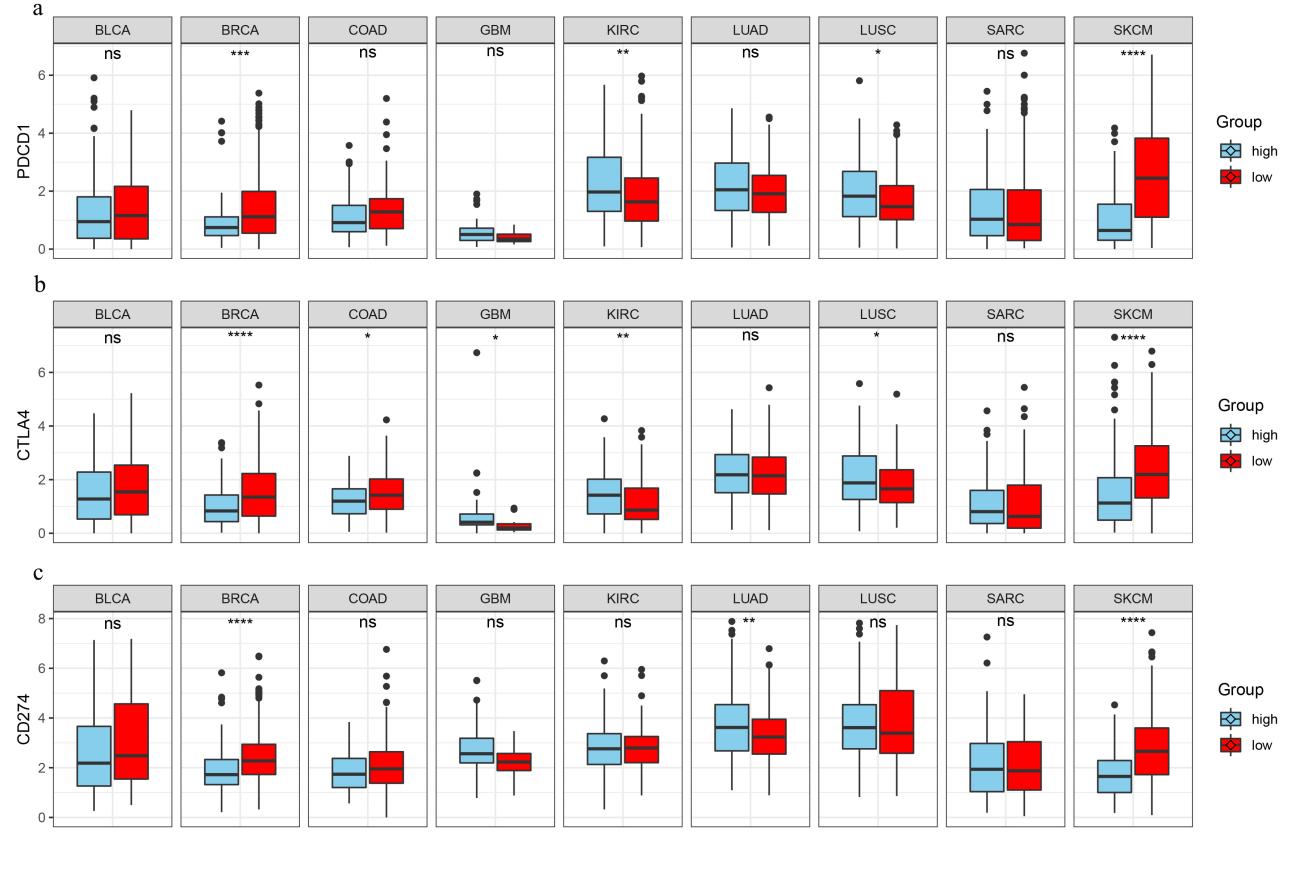


**Figure. S1** Box plots of high and low silencing score group differences in immune checkpoints of PDCD1 **(a)** ,CTLA4 **(b)** and CD274 **(c)**.


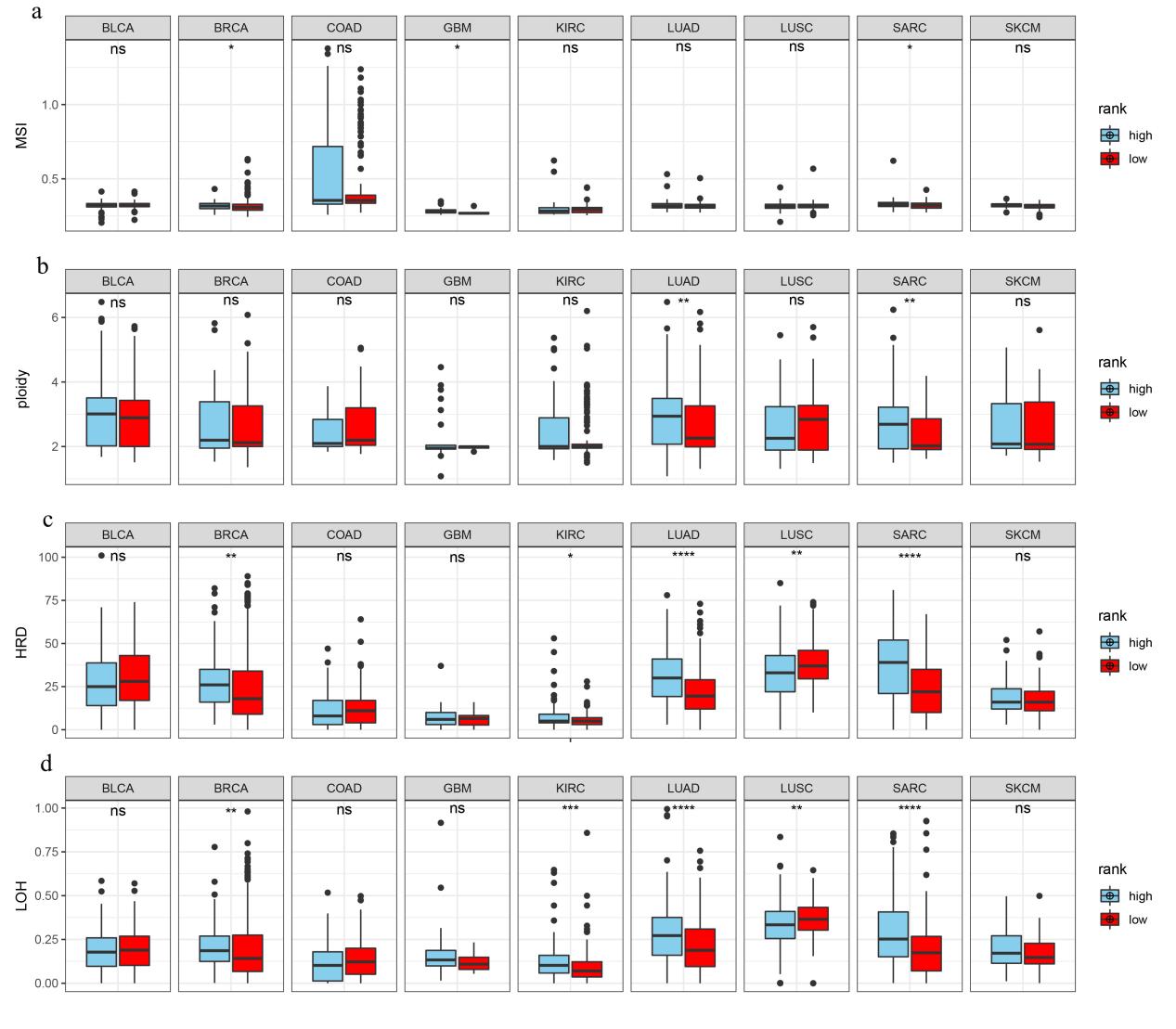


**Figure. S2** Levels of a list of genome instability scores among two groups. This list includes MSI **(a)**, aneuploidy **(b)**, HRD **(c)**, and LOH **(d)**.

MSI, Microsatellite Instability; LOH, loss of heterozyosity; HRD, homologous recombination deficiency


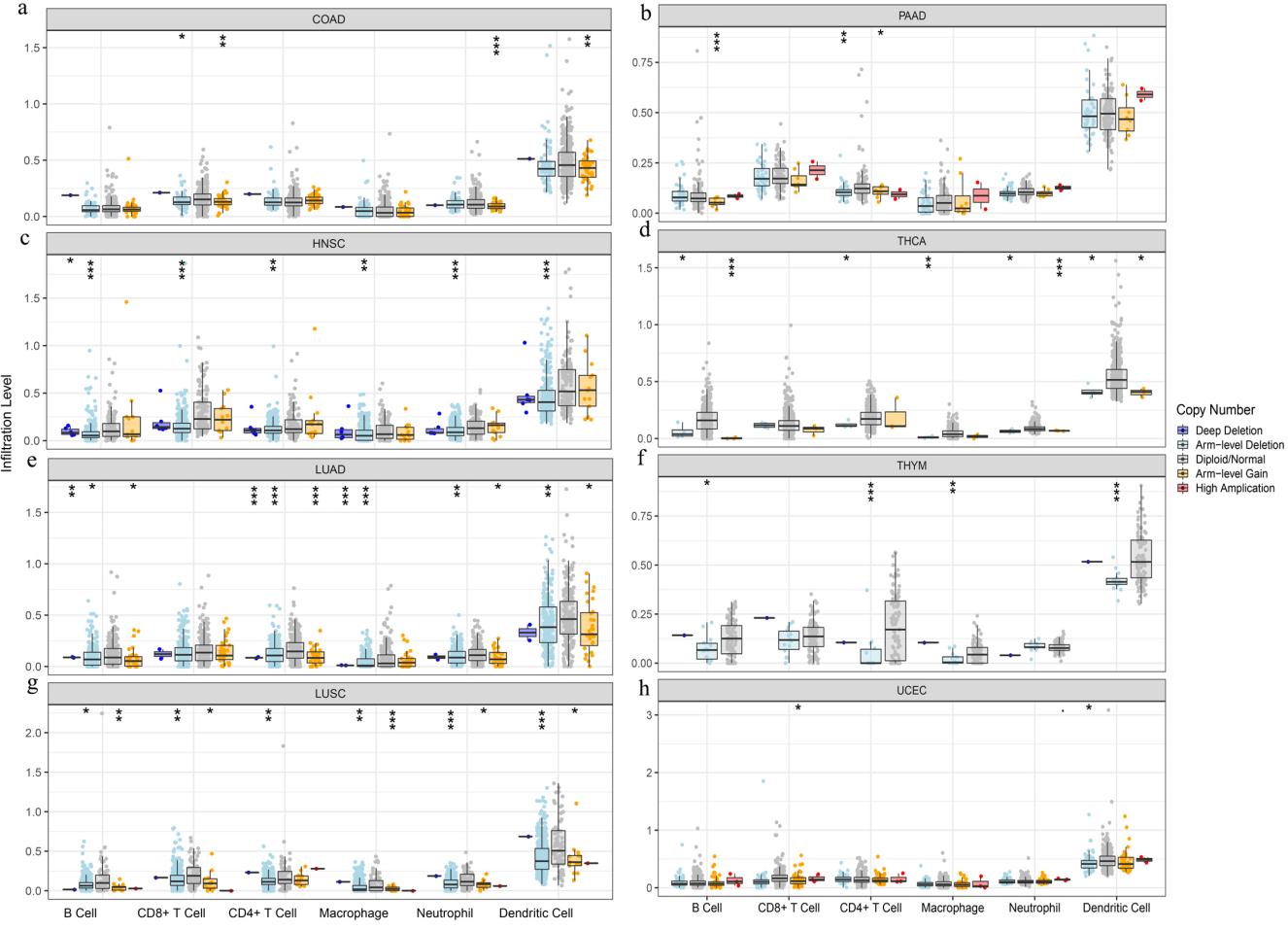


**Figure. S3** The differences of tumor immune infiltration across deep deletion, arm-level deletion, arm-level gain and high amplication, compare with normal status. COAD, Colon adenocarcinoma; PAAD, Pancreatic adenocarcinoma; HNSC, Head and Neck squamous cell carcinoma; THCA, Thyroid carcinoma; LUAD, Lung adenocarcinoma; THYM, Thymoma; LUSC, Lung squamous cell carcinoma; UCEC, Uterine Corpus Endometrial Carcinoma.
